# Dissection of genetic and environmental factors involved in tomato organoleptic quality

**DOI:** 10.1186/1471-2229-11-58

**Published:** 2011-03-31

**Authors:** Paola Carli, Amalia Barone, Vincenzo Fogliano, Luigi Frusciante, Maria R Ercolano

**Affiliations:** 1 Department of Soil, Plant, Environmental and Animal Production Sciences, University of Naples 'Federico II', Via Universita' 100, 80055 Portici (NA), Italy; 2 Department of Food Science, University of Naples 'Federico II', Via Universita' 133, 80055 Portici (NA), Italy

## Abstract

**Background:**

One of the main tomato breeding objectives is to improve fruit organoleptic quality. However, this task is made somewhat challenging by the complex nature of sensory traits and the lack of efficient selection criteria. Sensory quality depends on numerous factors, including fruit colour, texture, aroma, and composition in primary and secondary metabolites. It is also influenced by genotypic differences, the nutritional regime of plants, stage of ripening at harvest and environmental conditions. In this study, agronomic, biochemical and sensory characterization was performed on six Italian heirlooms grown in different environmental conditions.

**Result:**

We identified a number of links among traits contributing to fruit organoleptic quality and to the perception of sensory attributes. PCA analysis was used to highlight some biochemical, sensory and agronomic discriminating traits: this statistical test allowed us to identify which sensory attributes are more closely linked to environmental conditions and those, instead, linked to the genetic constitution of tomato. Sweetness, sourness, saltiness and tomato flavour are not only grouped in the same PCA factor, but also result in a clear discrimination of tomato ecotypes in the three different fields. The three different traditional varieties cluster on the basis of attributes like juiciness, granulosity, hardness and equatorial diameter, and are therefore more closely related to the genetic background of the cultivar.

**Conclusion:**

This finding suggests that a different method should be undertaken to improve sensory traits related to taste perception and texture. Our results might be used to ascertain in what direction to steer breeding in order to improve the flavour characteristics of tomato ecotypes.

## Background

Tomato consumers are becoming increasingly demanding as regards the external appearance, nutritional and organoleptic characteristics of fruits. In addition to nutritional quality, sensory quality (i.e. visual aspect, firmness, and taste) is of utmost importance for fruit consumption. Although visual appearance is a critical factor driving initial consumer choice, in subsequent purchases eating quality becomes the most influential factor [1]. To satisfy consumer expectations, tomato breeders are now pursuing sensory quality as one of their major breeding objectives, although the complex nature of many of the sensory traits and the lack of efficient selection criteria make it a difficult task.

Sensory quality depends on numerous factors, including fruit colour, texture, aroma, and composition in primary (sugars, organic acids and amino acids) [2-4] and secondary metabolites [5-7]. Several studies have established that the organoleptic quality of tomato for fresh consumption is conditioned mainly by the increase in organic acids and carbohydrates [8,9]. Indeed, a balanced sugar/organic acid ratio was preferred by a panel examining the flavour characteristics of cherry tomato [10]. Free amino acids may play the role of taste-enhancement [11,12], with glutamic acid the main free amino acid present in tomatoes [13]. The concentration levels of these molecules may significantly affect tomato flavour acceptability [8]. Several studies have been performed to identify associations between biochemical or physical fruit characteristics and sensory traits [14-16]. QTLs that control the variation of sensory and biochemical traits and the composition of volatile chemicals contributing to overall fruit flavour have been identified [12,17,18] and used to assist selection [19]. Ercolano *et al. *[20] provided a compendium of information, including phenotypic, biochemical and molecular data, on traditional tomato ecotypes that could constitute the basis to elucidate which biochemical factors are mainly involved in tomato fruit flavour determination. Network analysis was able to reduce data complexity by focusing on key information of the full data set. A number of links among traits contributing to fruit organoleptic quality and to the perception of sensory attributes were identified [21].

In order to gain a clearer understanding of the biochemical and genetic control of the generation of flavour compounds in tomato, the objectives of this work were: 1) to assess flavour diversity of six Italian ecotypes grown in different environmental conditions; 2) to identify important correlations among biochemical and sensory components affecting tomato flavour; 3) to separate traits that depend on genetic constitution from those that interact more with the environment.

## Results

In order to evaluate tomato organoleptic quality, agronomic, biochemical and sensory analyses were performed on ripe fruits of six local ecotypes harvested in three different fields.

### Biochemical analysis

The results obtained from physicochemical and biochemical analysis performed on tomato fruits (Table 1) reveal profound differences between the lines in the levels of several metabolites. The pH value ranged from 3.82 (100 SCH in the Ercolano field) to 4.60 (SOR ADG in Sorrento). The highest pH values were detected in all tomato ecotypes harvested in the Sorrento field while the lowest in samples harvested in Ercolano. By contrast the °Brix value, ash and dry matter were significantly higher in all samples grown in Ercolano, where almost 70% of the samples scored dry matter >8. Interestingly, genotype VES 2001, in all fields, was the ecotype with the highest dry matter. Significant differences between single ecotype harvests in different fields were found for these traits.

**Table 1:** Evaluation of physicochemical and biochemical traits of fruit from six tomato ecotypes grown in three different fields.

Ercolano									
**Ecotypes**	***pH***	***°Brix***	***Ash (%)***	***Dry Matter (%)***	***Malic Acid***	***Ascorbic Acid***	***Citric Acid***	***Fumaric Acid***	***Total amino acids***
					
					**mg per 100 g of fresh weight**				

SM. Sch.	3.90 ± 0.14	6.80 ± 0.00	0.77 ± 0.01	7.78 ± 0.08	82.4 ± 13.5	5.48 ± 0.03	435 ± 2.6	0.20 ± 0.03	241 ± 35.6
SM Sel. 8	4.09 ± 0.06	7.00 ± 0.20	0.63 ± 0.01	8.35 ± 0.12	41.8 ± 5.61	1.49 ± 0.04	432 ± 7.7	0.20 ± 0.03	219 ± 27.3
Sor. Adg.	3.97 ± 0.06	6.17 ± 0.06	0.65 ± 0.01	5.83 ± 0.08	52.0 ± 4.18	0.00 ± 0.00	489 ± 2.3	0.08 ± 0.00	552 ± 32.5
Sor. Art.	3.86 ± 0.08	7.73 ± 0.11	0.72 ± 0.03	8.08 ± 0.34	79.5 ± 0.60	6.20 ± 0.21	702 ± 3.6	0.26 ± 0.01	136 ± 8.1
Ves. 2001	3.83 ± 0.06	7.90 ± 0.10	0.84 ± 0.01	10.5 ± 0.35	224 ± 18.6	1.83 ± 0.09	597 ± 11.1	0.29 ± 0.04	196 ± 16.4
100 Sch.	3.82 ± 0.14	7.33 ± 0.31	0.82 ± 0.01	9.51 ± 0.27	87.6 ± 7.51	1.13 ± 0.01	575 ± 9.2	0.13 ± 0.06	241 ± 22.7

**Sorrento**									

SM. Sch.	4.36 ± 0.09	5.73 ± 0.11	0.67 ± 0.02	6.19 ± 0.12	13.5 ± 1.15	0.00 ± 0.00	265 ± 3.11	0.22 ± 0.03	469 ± 43.6
SM Sel. 8	4.25 ± 0.05	5.00 ± 0.00	0.60 ± 0.03	6.73 ± 0.24	35.4 ± 1.57	1.52 ± 0.04	317 ± 13.7	0.17 ± 0.04	485 ± 32.6
Sor. Adg.	4.60 ± 0.04	4.27 ± 0.23	0.42 ± 0.02	4.45 ± 0.14	66.7 ± 9.06	0.69 ± 0.03	228 ± 7.15	0.11 ± 0.01	407 ± 27.8
Sor. Art.	4.23 ± 0.29	4.60 ± 0.34	0.46 ± 0.00	5.47 ± 0.12	55.7 ± 4.85	1.80 ± 0.04	314 ± 0.77	0.34 ± 0.01	184 ± 12.7
Ves. 2001	4.05 ± 0.24	5.67 ± 0.30	0.72 ± 0.01	7.48 ± 0.27	86.7 ± 1.27	5.80 ± 0.00	292 ± 3.53	0.34 ± 0.02	94 ± 8.5
100 Sch.	4.35 ± 0.06	5.07 ± 0.11	0.63 ± 0.01	6.45 ± 0.39	136 ± 5.84	6.74 ± 0.05	320 ± 0.87	0.55 ± 0.04	211 ± 14.6

**Sarno**									

SM. Sch.	4.30 ± 0.07	4.80 ± 0.34	0.40 ± 0.01	5.47 ± 0.41	24.1 ± 2.72	1.49 ± 0.06	278 ± 1.71	0.10 ± 0.00	1100 ± 47.3
SM Sel. 8	4.07 ± 0.20	5.27 ± 0.23	0.56 ± 0.01	6.00 ± 0.00	86.0 ± 15.0	0.00 ± 0.00	251 ± 2.96	0.21 ± 0.00	953 ± 24.4
Sor. Adg.	4.25 ± 0.18	5.40 ± 0.40	0.43 ± 0.01	6.49 ± 0.51	25.7 ± 2.43	0.34 ± 0.01	338 ± 6.25	0.00 ± 0.00	389 ± 9.5
Sor. Art.	4.09 ± 0.19	5.60 ± 0.34	0.53 ± 0.02	6.56 ± 0.04	86.1 ± 4.85	3.04 ± 0.11	435 ± 0.10	0.22 ± 0.04	852 ± 15.7
Ves. 2001	4.01 ± 0.05	6.53 ± 0.23	0.51 ± 0.00	8.75 ± 0.11	129 ± 5.06	6.59 ± 0.04	425 ± 7.25	0.25 ± 0.00	760 ± 27.6
100 Sch.	4.10 ± 0.21	5.20 ± 0.20	0.46 ± 0.00	6.17 ± 0.05	60.1 ± 6.42	0.46 ± 0.00	333 ± 0.60	0.24 ± 0.01	1345 ± 37.5

Values are presented as mean ± SD of two independent determinations.

As regards organic acids, citric acid reached high concentrations in all samples, though displaying significant variability (P < 0.01) between the different fields, ranging from 702.7 mg 100 g^-1^in SOR ART in Ercolano to 228 mg 100 g^-1^in SOR ADG in Sorrento. In samples harvested in Ercolano citric acid content was always quite high, with values exceeding 400 mg 100 g^-1^. Genotypes VES 2001 and SOR ART reached concentrations in excess of 600 mg 100 g^-1^. With regard to malic acid contents, great variations were observed in single sample harvests in different fields. For instance in SM SCH the concentration of this acid varied from 13 mg 100 g^-1 ^in Sorrento to 8.2 mg 100 g^-1 ^in Ercolano. Ecotype VES 2001 in Ercolano showed the highest concentration in malic acid (224 mg 100 g^-1^) while the lowest (13.5 mg 100 g^-1^) was found in SM SCH harvested in Sorrento. As for the concentrations of total free amino acids, the highest levels for all ecotypes were detected in Sarno (except for SOR ADG), the lowest in Ercolano (except for SOR ADG and 100 SCH). 100 SCH grown in Sarno was the sample with the highest concentration (1345 mg 100 g^-1^) while 100 SCH in Ercolano showed the lowest concentration (94 mg 100 g^-1^). Significant differences between the three fields were found in relation to Gln, Ser (P < 0.05), Asn, and Glu (P < 0.01) content. Furthermore, the data reveal that the main amino acid in all samples was glutamic acid, with values ranging from 982 mg 100 g^-1 ^in 100 SCH grown in Sarno to 39.6 mg 100 g^-1^in VES 2001 grown in Sorrento. Amino acids Asn and Gln were also found in quite high concentrations, with higher average values in Sarno. By contrast, Ser was completely absent in the VES 2001 ecotype harvested in all three fields.

### Agronomic analysis

**Figure 1:**
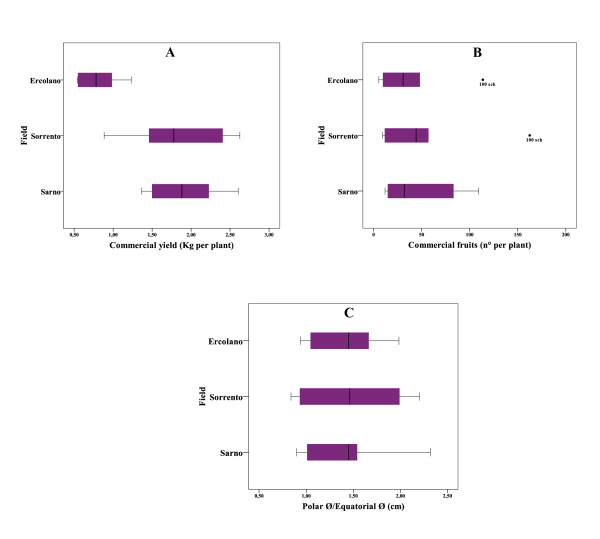
**A, B, C, Box plots of the agronomic data of fruit from six tomato ecotypes grown in three different fields, showing variation within single fields**. **A**, diagram of commercial yield, expressed as kg per plant, of six tomato ecotypes clustered into three different fields. **B**, diagram of commercial fruit expressed as no. of fruit per plant of six tomato ecotypes clustered into three different fields. **C**, ratio of polar and equatorial diameter of fruit per plant of six tomato ecotypes clustered into three different fields.

With regard to the agronomic evaluation performed on the ecotypes, statistical analysis (Figure 1) indicated that the genotype factor had a significant effect (P < 0.01) on the number of commercial fruits and polar/equatorial diameter whilst the three fields were statistically significant (P < 0.01) for marketable yield. On average, the commercial yield showed higher values in Sorrento (SOR ART: 2.63 kg per plant) followed by Sarno (100 SCH: 2.61 kg per plant) and last of all the Ercolano field where the lowest marketable yield was recorded (Figure 1A). As for the number of commercial fruit per plant (Figure 1B), there were huge differences between the field in Ercolano (lowest value) and Sorrento and Sarno which followed a similar trend. In particular, the two Sorrento ecotypes showed the lowest fruit number, following by the two San Marzano ecotypes and then by Vesuvio ecotypes. In detail, 100 SCH was the cultivar showing the highest fruit number with 162, 113 and 109 fruits recorded in the fields in Sorrento, Sarno and Ercolano, respectively. Finally, for the ecotypes grown in Sorrento the highest values of polar/equatorial diameter were observed, unlike the Sarno field where the lowest values for this trait were recorded (Figure 1C). With reference to the single ecotypes, as expected the two San Marzano cultivars had the best ratio in question while the two Sorrento cultivars presented the lowest polar/equatorial diameter.

### Sensory analysis

**Figure 2:**
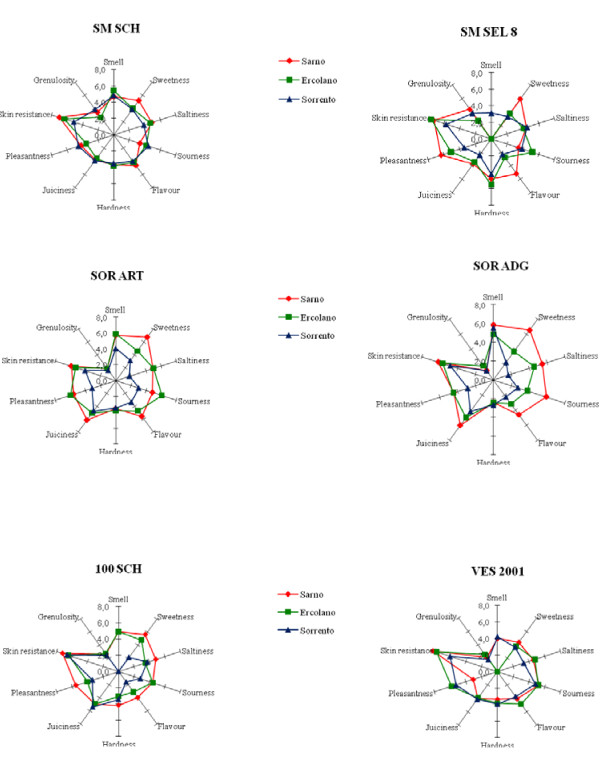
**Quantitative descriptive analysis of sensory attributes of the six tomato ecotypes grown in three different fields**. Individual attributes are positioned like the spokes of a wheel around a centre (zero, or not detected) point, with the spokes representing attribute intensity scales, with higher (more intense) values radiating outward. Legend: red is used for the tomato ecotypes grown in the Sarno field; green, the tomato ecotypes grown in Ercolano; blue, the tomato ecotypes grown in Sorrento.

A sensory test was conducted to characterise the properties of tomato fruit by means of quantitative descriptive analysis (QDA). Spider plots were created by plotting average intensity values on each scale, and then joining the points. Results of the sensory tests on the ecotypes harvested in the three different fields are shown in Figure 2. The profiles obtained through the panel test summarise the sensory attributes of the ecotypes analysed. The panel of trained assessors found significant differences in saltiness (P < 0.01), sourness (P < 0.01), sweetness (P < 0.01) and skin resistance (P < 0.05) for the three different fields. Single genotypes, instead, showed significant differences in hardness (P < 0.01), juiciness (P < 0.01) and granulosity (P < 0.01).

The plots illustrated that all ecotypes harvested in Sarno displayed the most intense flavour attributes and sweetness. The samples harvested in Sorrento had marked acidity while the Ercolano ecotypes showed low acidity and intermediate sourness. In general, all the ecotypes grown in Ercolano were given the lowest attribute intensity, those grown in Sorrento intermediate intensity and those in Sarno the highest intensity, for all traits evaluated.

Considering single sample data, some traits peculiar to each type were evidenced. The two San Marzano ecotypes (SM SCH and SM Sel. 8) showed higher granulosity than the others, whereas for juiciness, the most intensity was found in the two Sorrento ecotypes (SOR ART and SOR ADG) in all three fields. The two Sorrento ecotypes also showed the lowest intensity of granulosity in all fields. Moreover, SOR ADG in Sarno received higher scores for taste attributes (sweet, sour and salt).

### Correlation and PCA analysis

**Figure 3:**
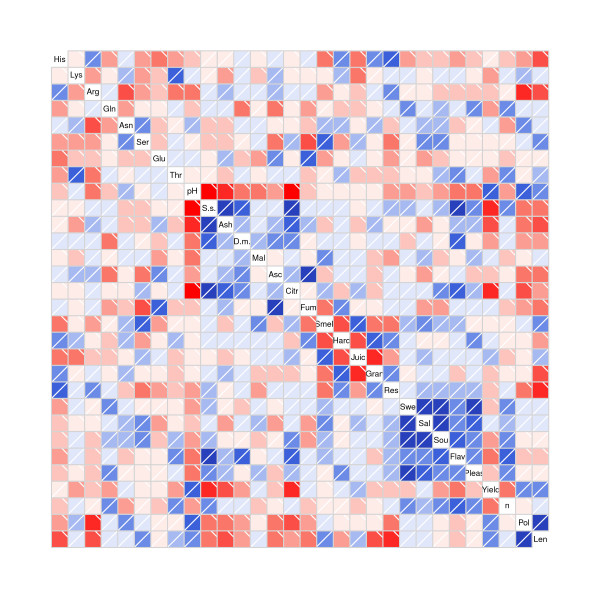
**Heat map showing correlation analysis among physicochemical, biochemical, sensory and agronomic traits in six tomato ecotypes grown in three different fields**. Regions in red and blue indicate negative or positive correlations among the traits, respectively. Abbreviations: His, Histidine; Lys, Lysine; Arg, Arginine; Gln, Glutamine; Asn, Asparagine; Ser, Serine; Glu, Glutamic acid; Thr, Threonine; pH, pH, SS., Soluble solid; Ash, Ash; DM., Dry Matter; Mal, Malic acid; Asc, Ascorbic acid; Citr, Citric acid; Fum, Fumaric acid; Smell, Tomato smell; Hard, Hardness; Juic, Juiciness; Gran, Granulosity; Res, Skin resistance; Swe, Sweetness; Sal, Saltiness Sou, Sourness; Flav, Tomato flavour; Pleas, Pleasantness; Yield, Commercial yield; n, Number of commercial fruits; Pol, Polar diameter; Len, Equatorial diameter.

For a fuller characterization of the associations between traits evaluated, a correlation-based approach was adopted using the Pearson coefficient as an index of correlation. The heat map (Figure 3) shows the correlations between metabolites and sensory properties. In all, 435 correlations between biochemical, sensory and agronomic traits were detected. Of these correlations, 229 were positive and 206 were negative. Furthermore, 86 correlations were significant with a significance level of 0.05. In particular, three major correlation groups with a large number of internal links were observed. The first group comprised the strong negative links among the pH and other biochemical traits and strong positive links among physicochemical and biochemical parameters. The second group included the connections (some positive and some negative) among the sensory attributes responsible for tomato texture, such as tomato juiciness, granulosity, hardness and skin resistance. The attributes belonging to the taste group (sweetness, sourness, saltiness, tomato flavour and pleasantness) showed strong positive correlations among themselves: tomato flavour is strongly negatively related with soluble solid, ash, dry matter and citric acid. Finally, the agronomic traits showed numerous links, among themselves and among biochemical and sensory characteristics. Indeed, fruit yield and polar diameter seem more correlated with biochemical traits, whilst equatorial diameter proved more correlated with sensory attributes (tomato smell, juiciness, granulosity, hardness and skin resistance).

Principal component analysis was carried out on the agronomic, biochemical and sensory traits to describe relations among the different attributes as well as detect important components. Six principal components were obtained that explained approximately 80.3% of the variability in the dataset. The first two factors explained about 38% of the variation in the data, with the first component alone (PC1) accounting for more than 23% of the variation and the second component (PC2) accounting for 15% of the variation. The first factor was strongly associated with Lys amino acid, physico-chemical parameters (pH, soluble solids, dry matter, and ash), with citric acid and commercial yield, while factor 2 was mainly associated with sensory traits such as sweetness, sourness, saltiness, pleasantness and tomato flavour and with the amino acid Gln. By contrast, the third factor (14%) was dominated by juiciness, granulosity and hardness, and by equatorial diameter.

The fourth factor accounts for a further 11% of the variability, and consists in the Asn, Ser, Glu and Thr amino acids, and in skin resistance. The fifth and sixth factors explained 11% and 8% of total variability, respectively. The fifth was associated with Arg amino acid, ascorbic and fumaric acids, and two agronomic traits, fruit number and polar diameter, while the sixth was dominated by two biochemical traits (His amino acid and malic acid) and one sensory attribute (tomato smell). Plotting the factor scores as coordinates on the axes of two- or three-dimensional scatter plots, a graphical representation of the relationship between samples in a PCA was generated. In this study several two-dimensional scatter plots were generated for each dataset using component pair combinations from the seven principal components.

**Figure 4:**
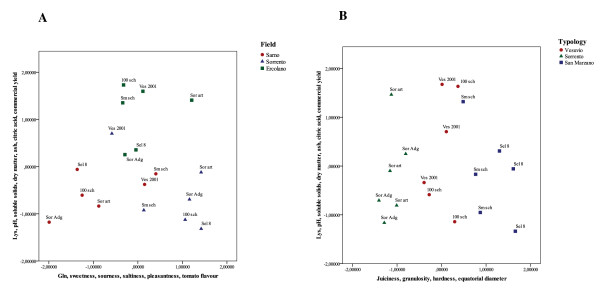
**Principal component analysis of the physicochemical and biochemical compounds, agronomic traits and sensory attributes, in tomato ecotypes harvested in three different fields**. Axes of two-dimensional plots are derived from (A) PC-1 and PC-2, (B) PC-1 and PC-3. These factors were chosen for the best visualization of field and genotype separation and include 50% of the total information content. Plotted points represent individual samples. In scatter plot A different coloured points were used to indicate samples belonging to a same field. In scatter plot B different coloured points were used to indicate samples belonging to the same tomato type.

In Figure 4 all samples are represented as a function of factors PC1 and PC2, and PC1 and PC3. Figure 4A shows the two-dimensional principal component score plot using the first two score vectors, PC1 and PC2, which account for most variation. These two factors allowed us to cluster and separate samples in the three different fields on the basis of physicochemical parameters and some sensory attributes. As one would expect, ecotypes harvested in Ercolano were positioned in the upper-central part of the PC1 axis as they showed higher values for °Brix, dry matter and ash traits, while the PC2 factor determined the location of Sarno ecotypes in the lower left-hand part and those of Sorrento in lower right-hand part of the graphic. Instead, the PCA plots obtained by combining PC1 with PC3 (Figure 4B) allowed us to divide the genotypes into the three different types on the basis of their genetic constitution. The two ecotypes belonging to the San Marzano type are grouped on the right, the two Sorrento on the left and, finally, Vesuvio in the central part of the graphic.

## Discussion

Tomato breeders have expended considerable efforts trying to develop cultivars with improved fruit taste. However, many efforts have failed due to the complex interactions among the various biochemical components of tomato fruits, plants and fruit sensory characteristics. Indeed, tomato flavour is defined by a wide range of interactions among several physicochemical and sensory parameters and is influenced by plant nutritional regime [9], stage of ripening at harvest [22], genotypic differences and environmental conditions [23]. In this study, biochemical and sensory approaches were used to describe the phenotypic variation of a range of primary metabolites and sensory attributes across six different tomato ecotypes. Fruit components affecting tomato flavour were analysed and differences among traditional Italian varieties (San Marzano, Sorrento and Vesuvio) were highlighted. Most of the traits analysed, including some of the sensory attributes (saltiness, sourness and sweetness), varied greatly with environmental conditions. Such variations could be the result of different adaptations to field conditions among different ecotypes. On the other hand, for sensory traits such as juiciness, granulosity and hardness we found that varietal differences affected fruit quality more than growing conditions. Interestingly, our sensory analysis showed that such texture attributes obtained similar scores for the single genotypes independently of field location.

Understanding which ecotype characteristics could influence such attributes might be useful to identify which processes underlie these traits and their relationships, at both the genetic and physiological levels. As most of these quality traits are polygenically inherited, fruit parameters associated with sensory texture attributes were evaluated in order to gain knowledge concerning their genetic control [24]. The vast majority of correlations found in the present work (the strong positive links among the physicochemical and biochemical traits or among the taste attributes) supported the results obtained in our previous work [21]. Indeed, pH, dry matter and °Brix are highly correlated among themselves, and sensory attributes such as sweetness, saltiness and sourness for taste, and hardness, juiciness, granulosity and skin resistance for texture, did not show high connectivity with biochemical traits. However, it seems likely that considerable research effort is still needed in order to identify the cause, if any, underlying these relationships.

Principal component analysis (PCA) was applied to the combined sensory, biochemical and agronomic data to determine their relationships. PCA identified patterns of correlation showing the factor loadings and the relative positions among the products in a map. In particular, in our work, PCA analysis identified several biochemical, sensory and agronomic discriminating traits. They included: the amino acids Lys and Gln, physicochemical parameters such as dry matter, °Brix, ash and citric acid (factor 1), and taste attributes such as sweetness, sourness, saltiness, and tomato flavour (factor 2); texture attributes, namely juiciness, granulosity and hardness, and equatorial diameter (factor 3).

In particular, PCA allowed us to identify which sensory attributes are more influenced by environmental conditions and, those, instead, by the genetic constitution of tomato. Sweetness, sourness, saltiness and tomato flavour are not only grouped in the same factor (PCA-plot 1), but also produce a clear discrimination of tomato ecotypes in the three different fields.

While flavour traits such as sweetness and sourness are usually described on the basis of sugar and acid content, other external and internal stimuli can also regulate fruit taste perception. Despite advances in tomato flavour analysis, breeders and molecular biologists still lack a clear genetic target for selection and manipulation of tomato taste attributes [25-27]. Transcriptional regulation mechanisms can modify the expression level of highly responsive genes. In sugar and acid biosynthesis several mechanisms regulating expression or activity have been identified, such as compartmentalization breakdown and feedback regulation [28]. A genomic platform could facilitate the dissection of flavour traits to investigate the role of single genes as well as a gene network. For instance, silencing genes of interest can allow identification of key genes or regulatory elements in the flavour formation process.

In PCA-plot 2 the three different traditional variety types cluster together on the basis of attributes like juiciness, granulosity, hardness and equatorial diameter that are more related to the genetic background of cultivars. This finding suggests that genetic background had a greater impact on generating differences in texture profiles than environmental growth conditions. The genetic variation of such traits has been attributed to the joint action of many QTLs [29]. QTL analysis of fruit quality in fresh market tomatoes identified chromosome regions that control the physical and sensory variation of these traits [17,30]. However, slow progress has been made in improving such quantitative traits, due to several factors. First and foremost, the colocalizations of QTLs which create some antagonist effects, secondly the presence of several QTLs with low or less than additive effects [31] and finally also the interactions between QTLs and the environment or genetic background [24]. Dissecting complex traits into elementary physiological processes may help identify the genetic control of quality traits and in the search for candidate genes. It may be especially useful to screen NILs or mutant lines to seek the physiological processes involved in phenotypic variations [32,33]. Moreover development of fruit virtual models could help to narrow the gap between genes and complex phenotypes [34].

## Conclusion

In conclusion, biochemical and sensory profiling was performed in six tomato heirlooms grown in three different fields. The results confirmed and extended earlier studies [21], suggesting that environmental conditions and genetic background conditioned tomato fruit flavour. Although further studies will be required to grasp the complex factors underlying organoleptic quality in tomato, our results might be used to understand in what direction to steer breeding in order to improve flavour characteristics of tomato ecotypes. Tomato flavour improvement could be achieved by either traditional breeding techniques, modern biotechnology or a combination of both. A different method should be undertaken to improve sensory traits related to taste perception and texture. In the first case, approaches that allow modulation of the expression of genes involved in sugar and acid biosynthesis should be designed. In the second case, candidate genes should be identified and transferred into breeding lines. The emerging information on gene expression profiling during fruit ripening provides a basis for connecting genes and regulators with biochemical processes and hence a route for significant advances in breeding fruit crops fit for this purpose.

## Methods

### Plant material and growth

The materials used in this work comprised six different Italian tomato ecotypes: two Vesuvio ecotypes (100 SCH and VES 2001), two Sorrento (SOR ART and SOR ADG), and two San Marzano (SM SCH and SM Sel. 8). The genotypes were grown in randomised, replicated plots in three different sites in southern Italy (Sorrento, Sarno and Ercolano) during the summer of 2006. Young seedlings (~1 month old) were planted at the end of April in a randomized complete block design with two replications. Plants were grown under the standard tomato field procedures used in the area. Ripe fruits from all plants for each line were harvested three times, and fruit yield (kg per plant), number of fruits, and morphological traits (fruit polar and equatorial diameters) recorded for single plants. At the three different harvesting times, one sample per replicate (10 plants) of 2-6 kg was obtained by pooling fruits belonging to each genotype. Random pieces of fruits were used to conduct sensory evaluation. The fruits were then homogenized, divided into aliquots, and stored at -20°C to determine chemical and biochemical parameters.

### Chemicals

All solvents used for HPLC analysis were purchased from Merck (Darmstadt, Germany). The malic and fumaric acid standards were from ICN Biomedical Inc., ascorbic acid and citric acid were from Sigma (CA, St Louis, MO, USA), and the amino acids were supplied by Bachem (Switzerland).

### Metabolic analysis

In order to perform physical, chemical, and biochemical analyses, a homogenized mix of fruits was obtained from the three field harvests of each genotype. The following parameters were determined on all samples in duplicate: pH at 20°C (HI 9017 Microprocessor pHmeter, Hanna Instruments), refractive index at 20°C (°Brix), total solids, ash, organic acids, and amino acids. The soluble solid concentration in the fruit was estimated by means of the Brix degree, determined on the homogenate by an RFM330 Refractometer (Bellingham Stanley Ltd, UK). Total solids (dry matter content) were estimated by drying 5 g of fresh fruit in an oven (Ehret) set at 70°C until constant weight was reached. Results were expressed as percentages of fresh weight. Ash content was calculated from the weight of the sample after burning at a temperature of 105°C overnight [35].

### Organic Acids

The organic acids (malic, citric, ascorbic and fumaric) were determined by HPLC analysis. Briefly, 0.1 g of lyophilized sample were added to 5 ml of H_2_SO_4_/H_2_O 0.008 N, agitated for 1 min. and centrifuged at 4000 rpm for 5 min at 4°C. Two ml of the supernatant were collected and centrifuged at 12000 rpm for 2 min at 4°C. An aliquot of the extract was used for analysis by HPLC configured with LC-10AD pumps, SLC10A system control, diode array UV-VIS detector (Shimadzu Japan) and Synergy Hydro column (4 μm, 250 mm × 4.6 mm; Phenomenex). The organic acids were eluted with H_2_SO_4_/H_2_O 0.008 N at 1.0 ml/min flow under isocratic conditions at 210 nm for malic, citric and fumaric acids, and at 245 nm for ascorbic acid. Extraction was repeated twice for each sample. The data obtained were expressed as milligrams of organic acids per 100 g of fresh matter.

### Amino acids

In order to evaluate the amino acid content, 25 g of freeze-dried tomato samples were dissolved in 15 ml of deionized water and centrifuged at 4000 rpm for 15 min. The supernatant was filtered and centrifuged using a Centricon YM-3 (Millipore, USA). A 500 μl aliquot of filtrated sample was dried and dissolved in 500 μl of borate buffer (0.1 M, pH 10.4). The solution was mixed with FMOC reagent (500 μl, 5.8 mM in acetone) [36]. The mixture was extracted twice with 2 ml of hexane/ethyl acetate (80:20). The aqueous phase containing the FMOC derivatives was analysed by RP-HPLC interfaced with an ESI-MS (electrospray ionization-mass spectrometer; API-100 Sciex, Canada), using the following conditions for HPLC and MS.

HPLC: Liquid chromatography (LC) analyses were performed using two micro pump series 200 (Perkin Elmer; Canada). A Luna 5 μ C_18 _column, 250 × 4.6 mm (Phenomenex, USA) was used. Eluents were water 0.05% TFA (solvent A) and acetonitrile 0.05% TFA (solvent B). The FMOC derivates were separated using the following linear gradient: 30-50% B in 15 min, 50-100% B in 20 min, 5 min isocratic elution at 100% B. The LC flow rate was set at 0.8 mL/min and after split 50 μL/min were sent to the mass spectrometer. Injection volume was 50 μL.

MS: Analyses were performed using a single-quadrupole API 100 mass spectrometer equipped with an electrospray (ESI) source in positive mode. The operating parameters were as follows: capillary voltage (IS) 5000 V, orifice voltage (OR) 100 V. Acquisition was performed in SIM (single ion monitoring) using a dwell time of 300 ms.

### Sensory analysis

Sensory analyses were performed by a trained panel working in a sensory laboratory under defined (temperature and light) conditions in single cabins with computer equipment. The sensory panel comprised 10 judges, aged 20 to 50, who had previously been trained in the quantitative description of tomato attributes according to selection trials based on the ISO 8586-1:1997 [37]. In the week prior to the test sessions, the panelists participated in specific training sessions on the products (4 sessions of 90 min each). During the training sessions, panelists were presented with a variety of tomato samples representing different cultivars on characteristic tomato flavor. The panel leader compiled a descriptor list from published literature on tomato flavor to aid panelists in verbalizing flavor and aroma characters perceived in the samples. During the training sessions, panellists reached the consensus on 10 different attributes: one for smell (tomato smell), four for taste (sweetness, saltiness, sourness, pleasantness), one for flavour (tomato flavour) and four for texture (hardness, juiciness, granulosity, skin resistance). The intensity of sensory perception by the trained panel was determined twice for each type of product with the use of unstructured line scales with the anchor points 0--not perceptible, and 10--strongly perceptible.

### Statistical analysis

MANOVA analysis and principal component analysis were performed by using SPSS (Statistical Package for Social Sciences) Package 6 version 17.0. Results were analysed by analysis of variance with a significance of P < 0.01 and 0.01 < P < 0.05 in order to test the significance of the observed differences.

PCA was applied to describe the relations between the agronomic traits, biochemical compounds and sensory attributes. To facilitate interpretation of the results, the factors were orthogonally rotated (which leads to uncorrelated factors), following the 'Varimax' method. Principal component analysis (PCA) is a widely used multivariate analytical statistical technique that can be applied to data to reduce the set of dependent variables (i.e., attributes, traits) to a smaller set of underlying variables (called factors) based on patterns of correlation among the original variables [38].

## Authors' contributions

PC planned, conducted and analyzed most of the experiments and was centrally involved in writing the manuscript. AB helped to coordinate the project and edited the final manuscript. VF contributed to obtain the biochemical data. LF provided significant ideas and critical review of the manuscript. MRE conceived the overall project, analysed results and planned experiments, and was a primary author of the manuscript. All authors read and approved the final manuscript.
